# Importance of Local Data and Resource Allocation for Effective Successful Public Health Interventions to Reduce COVID-19 Transmission: Commentary on COVID-19 Medical Vulnerability Indicators: A Predictive Local Data Model for Equity in Public Health Decision-Making

**DOI:** 10.3390/ijerph18105454

**Published:** 2021-05-20

**Authors:** Edward Sondik, Jonathan Fielding

**Affiliations:** 1 National Center for Health Statistics, Hyattsville, MD 20782, USA; 2 Fielding School of Public Health, University of California, Los Angeles, CA 90095, USA; jfieldin@ucla.edu

The article, *COVID-19 Medical Vulnerability Indicators: Predictive Local Data Model for Equity in Public Health Decision-Making (2021)*, is an important contribution to identifying and prioritizing the needs of Los Angeles’ public healthcare in responding to the COVID-19 pandemic crisis [1]. The authors developed a framework for, in effect, disaggregating a diverse population’s complex vulnerabilities to COVID-19 and identifying subpopulations at different risks geographically. They used these different aspects of risk for COVID-19 to produce a framework for identifying how to allocate resources among interventions and subpopulations in order to achieve the most effective impact.

Ong, Mays, and their colleagues have shown the importance of detailed local data, that is, being able to divide the population into relatively small geographic areas with information on the demographics of the subpopulations, along with the factors that make the population vulnerable to COVID-19. The framework is based on four types of risk that can be displayed geographically, in effect partitioning Los Angeles County into a set of communities characterized by their demographics and by four risks (or vulnerabilities). This is an ideal framework for resource allocation decisions. Coupling this with the constraints of interventions, including their costs and limitations for materiel and healthcare workers, creates an analytic structure to inform decision makers. One reason the creative use of local data is particularly beneficial in Los Angeles is that one of the criteria for relaxing COVID induced restrictions relates to improvements in health equity within the overall county population. This state-imposed equity metric is designed to ensure that the test positivity rates in the most disadvantaged neighborhoods do not significantly lag behind the overall county positivity rate. Each county must submit a plan demonstrating targeted investments to eliminate disparities in the levels of COVID transmission. Local data are an essential to that task, both in plan submission and evaluation over time.

The crucial sources for detailed data invaluable for addressing COVID and many other population health risks and conditions are the American Community Survey and AskCHIS Neighborhood Edition, which can be analyzed by a geographic base of small areas—zip code tabulation areas. The data source on parks and open space is an important adjunct. The importance of local data is emphasized in recommendations on how to achieve the newly released objectives of Healthy People 2030 (HP 2030) [2], the fifth decade of a national program to set national health goals. Over the next decade, HP 2030’s data will be used to guide and evaluate progress in meeting the objectives. HP 2030’s Secretary Advisory Committee has emphasized the importance of having data that can be used at local levels to evaluate progress and identify barriers and challenges to effecting change [3]. It is at the local or community level where population change must be achieved in order to improve health outcomes.

This article’s local data-based framework need not be exclusive to California. In fact, the American Community Survey’s data are available, aggregated at the level of zip code tabulation areas with five-year estimates for most of the US population. This is an excellent start to address the HP 2030 objectives. However, Los Angeles is fortunate to have another key element in using local data as the basis for health interventions: a wide base of expertise in the use of data, mapping, linking interventions to measures of vulnerability, and maintaining this framework. The authors have made a major step forward and we look to our country building on this expertise. Furthermore, we look forward to research to meld this framework with the extensive ongoing COVID-19 modeling. These insights could be extremely helpful for bringing the pandemic under control.
